# Case Report: Pediatric Chronic Active Epstein-Barr Virus Infection With Giant Sinus of Valsalva Aneurysms and Aorta and Its Branch Dilations

**DOI:** 10.3389/fped.2021.779806

**Published:** 2022-01-31

**Authors:** Qirui Li, Guyu Li, Daming Shao, Tharak Yarrabolu, Yuan Yue

**Affiliations:** ^1^Department of Cardiology, Beijing Children's Hospital, Capital Medical University, National Center for Children's Health, Beijing, China; ^2^Department of Pediatrics, Driscoll Children's Hospital, Corpus Christi, TX, United States; ^3^Department of Medicine, Jacobi Medical Center, New York City, NY, United States; ^4^Department of Cardiology, Driscoll Children's Hospital, Corpus Christi, TX, United States

**Keywords:** Chronic active Epstein-Barr virus infection, sinus of Valsalva aneurysms, multiple artery aneurysms, systemic vasculitis, pediatrics–children

## Abstract

Chronic active Epstein-Barr virus infection (CAEBV), which often manifests as persistent infectious mononucleosis-like symptoms and can involve multiple organs, is a prolonged or reactivated status of primary EBV infection. Cardiovascular damage is one of the rare but severe complications correlated with poor prognosis among all CAEBV patients. Few published articles have demonstrated systemic arterial lesions involving branches of the aorta as cardiovascular complications. Herein, we report a rare pediatric case of CAEBV associated with giant sinus of Valsalva aneurysms and aorta and its branch dilations.

## Background

Epstein-Barr virus (EBV), which replicates in the epithelial cells in the oropharynx and is transmitted by oral secretions (1), is a member of the herpesvirus family. Primary EBV infection is common in children or adolescents. Most patients are asymptomatic or only have non-specific symptoms of upper respiratory tract infection, or they manifest as infectious mononucleosis (IM). Primary EBV infection could happen in the B cells in the oropharynx. After the primary infection, memory B cells will generate at the persistence of EBV within the body. In some patients without significant immunosuppression, EBV can be reactivated and result in persistent infection named chronic active Epstein-Barr virus (CAEBV) infection. Patients with CAEBV usually present with chronic or recurrent IM-like symptoms, and multiple organ systems can be involved. The main clinical manifestations include continuous or intermittent fever, hepatomegaly, splenomegaly, lymphadenopathy, and skin damage (2). About 18–60% of patients with CAEBV were reported to develop cardiovascular complications during the long-term follow-up period, and almost half of them experienced coronary artery lesions (CAL). Few patients with CAEBV could also manifest as systemic vasculitis that mainly involves the aorta and its major branches (3–5). Very few cases involving sinus of Valsalva aneurysms with very poor prognosis were reported (6–8). Here, we report a pediatric patient with CAEBV accompanied by giant sinus of Valsalva aneurysms and dilation of the aorta and its major branches. To the best of our knowledge, there has only been one case reported by Nakagawa et al. (8).

## Case Presentation

In March 2017, a 5-year-old female (body weight 19.5 kg, Chinese, Chaoxian ethnicity) who presented with prolonged intermittent fever and a giant right coronary Valsalva sinus aneurysm was admitted to our department. Nine months before this admission, in July 2016, she had a fever (T-max 38.5°C) that lasted for 5 days without any precipitating factors. The fever was accompanied by oral vesicles and mild facial edema, and there are no other pertinent findings on history or physical exams. She was brought to local hospital A and found to have elevated inflammatory markers, alanine aminotransferase (ALT), and cardiac enzymes. Her presentations at that time raised concern for IM, and serological tests specific for EBV were sent, which demonstrated positives of EBV-viral capsid antigen (VCA)-IgM and EBV-early antigen (EA)-IgG, negatives of EBV-VCA-IgG, and EBV-nuclear antigen (NA)-IgG. The load of EBV-DNA detected by real-time polymerase chain reaction (PCR) was 3.2 × 10^5^ copies/μg DNA in peripheral blood. Therefore, she was diagnosed with IM with liver injury. After treatment with ganciclovir, her fever and facial edema improved, EBV-VCA-IgM turned negative, and EBV-VCA-IgG and EBV-NA-IgG turned positive, and then she was discharged.

After discharge, the patient continued to have intermittent fever with a peak temperature of 38°C, accompanied by progressive facial edema. Monitoring of inflammatory markers showed persistently elevated C-reactive protein (CRP) of 2.4 mg/dl and white blood cell count (WBC) of 12.8 × 10^3^/μl. In January 2017 (3 months before admission to our department), the patient was admitted to hospital B for persistently elevated inflammatory markers and fever. Laboratory evaluation was significant for negative EBV-VCA-IgM, with positives of EBV-NA-IgG, EBV-VCA-IgG, and EBV-EA-IgG, and the load of EBV-DNA was 3.66 × 10^5^ copies/μg DNA. Autoimmune panels showed negative ANCA and ANA. Her echocardiography showed a widened aortic sinus, with the widest site measuring 33.4 mm. Additionally, there was widened left and right coronary artery inner diameter. Coronary artery computed tomography angiography (CCTA) showed bilateral coronary aneurysms and giant right coronary sinus aneurysm measuring 22 mm × 25 mm; no coronary-pulmonary artery fistula was found. Carotid ultrasonography showed a slightly thickened tunica media. Duplex ultrasonography of renal artery, superior vena cava, superior mesenteric artery, and aorta were all negative for aneurysm or dilation. Due to persistent fever and elevated inflammatory markers, a bone marrow biopsy was also obtained, which showed hyperplasic bone marrow without leukemic cells.

The patient was diagnosed with Kawasaki disease remission stage with giant coronary sinus aneurysm and EBV infection (due to a lack of clinical information, a *Z*-score was not obtained). Given the large total number of aneurysms and involvement of multiple branches, she was determined to be at elevated risks of coronary artery thrombosis. Thus, she was treated with antiviral therapy with intravenous ganciclovir, dual-antiplatelet therapy with aspirin and dipyridamole (as no currently available drug information about clopidogrel usage in children with Kawasaki disease in China), and systemic anticoagulation therapy with oral warfarin. Due to no improvement of facial edema and intermittent fever, she was transferred to our hospital. This patient had a contact history of tuberculosis 3 months prior to admission without other significant past medical history and family history. Physical examination was pertinent for non-pitting facial edema, generalized lymphadenopathy, and hepatosplenomegaly.

**Figure 1:**
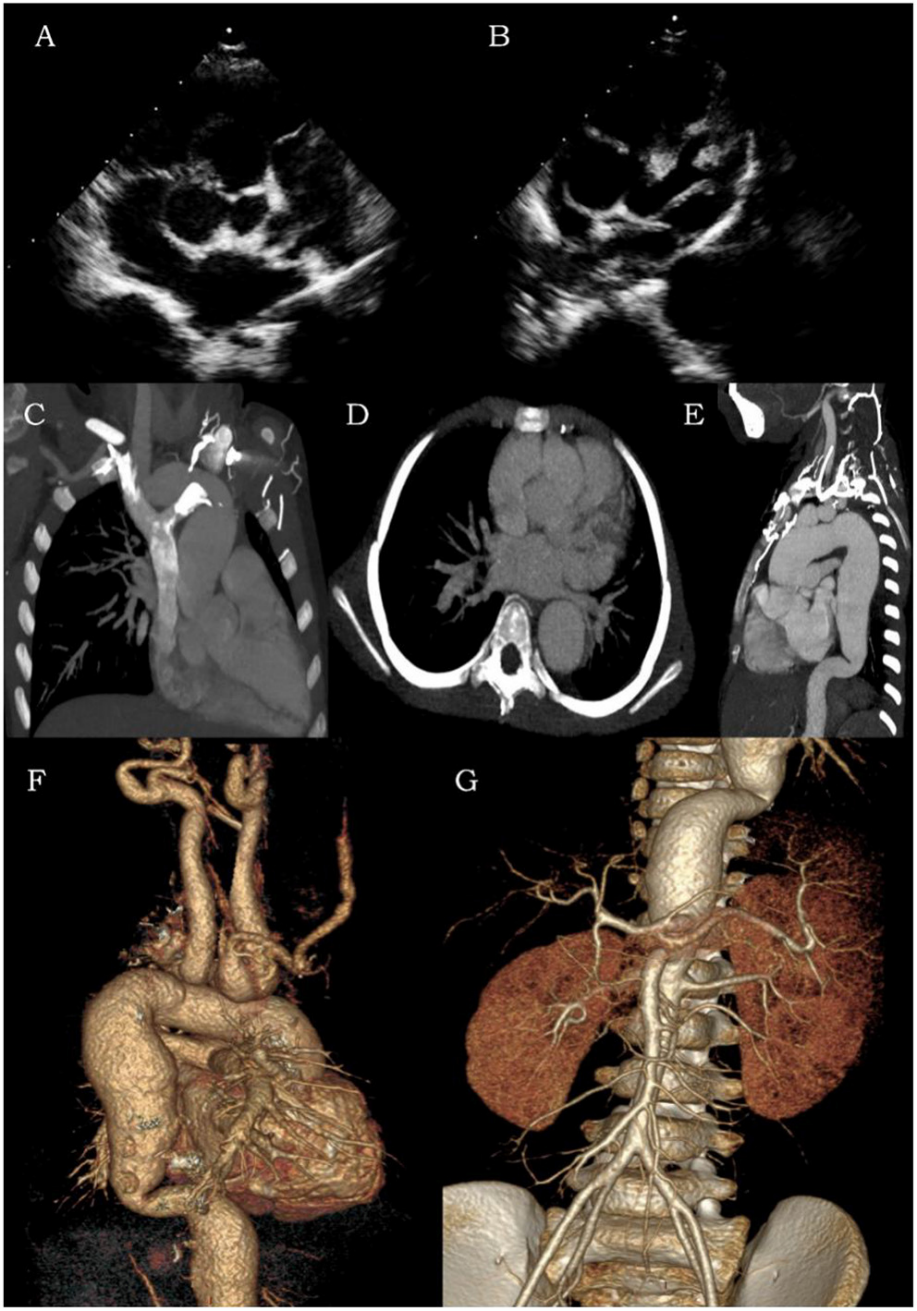
Transthoracic echocardiography **(A,B)** and computed tomographic angiogram **(C–G)** of coronary arteries, aorta, and its major branches. The contrast CT and color ultrasound showed multiple areas of arterial dilation in the aorta, coronary artery, bilateral carotid arteries, subclavian artery, brachiocephalic artery, innominate artery, and bilateral axillary arteries as well as compressed and narrowed jugular and left brachiocephalic veins with backflow obstruction.

On admission, the following laboratory results were obtained: elevated neutrophils of 55.6%, elevated CRP of 2.47 mg/dl, elevated AST of 93 U/L, and ALT of 67.8 U/L. Other serum values included WBC that was within normal limits. EBV serologic tests revealed negative EBV-VCA-IgM and positives EBV-VCA-IgG, EBV-EA-IgG, and EBV-NA-IgG. The load of EBV DNA was 1.05 × 10^5^ copies/μg DNA. Her ECG showed a second-degree atrioventricular block. Transthoracic echocardiography (TTE) revealed moderate mitral valve and tricuspid regurgitation, mild aortic regurgitation, giant sinus of Valsalva (SoV) aneurysms, particularly of the right coronary sinus (RCS) (Figure 1A), and a left main coronary artery (LMCA) aneurysm with the broadest diameter being about 7.9 mm (Figure 1B). A further computed tomographic angiogram (CTA) was obtained, which demonstrated multiple arterial aneurysms involving SoV, as well as LMCA (Figures 1C,D), aorta, and its major branches (Figures 1E–G). Dilated aneurysms could lead to venous hypertension with severe inflow restriction, and thus, multiple collateral vessels were produced. A lymph node biopsy for histopathological evaluation of EBV-infected cells was recommended but declined by the patient's family.

Based on her clinical manifestations, increased EBV-DNA, and positive EBV antibodies, we diagnosed this patient with CAEBV with multiple giant coronary artery aneurysms. Given the risk stratification as above, she was given 8 days of dual-antiplatelet therapy (aspirin 50 mg daily, dipyridamole 25 mg TID), systemic anticoagulation (warfarin 1 mg daily), and myocardial protection therapy (metoprolol 6.25 mg Q12H, captopril 6.25 mg Q12H). Spironolactone 5 mg Q12H and hydrochlorothiazide 6.25 mg Q12H were added for 2 days. Due to the parents' request, she was discharged after her parents were informed of discharge risks, and outpatient follow-up was scheduled to monitor disease progression, coronary artery aneurysms' size, and possible myocardial ischemia. Unfortunately, the patient did not follow up and died at home 15 months after the onset of the disease for unknown reasons. The clinical course is summarized in Table 1.

**Table 1:** Clinical courses.

	**Hospital A 9 months before admission**	**Hospital B 3 months before admission**	**Hospital C Current admission**
**Clinical features**			
T-max (°C)	38.5	37.3	38
Prolonged fever (days)	8	14	2
Lymphadenopathy	+	+	+
Hepatosplenomegaly	+	+	+
Facial edema	+	++	+++
**Lab results**			
WBC (× 10^3^/μl)	12.5	Unknown	4.86
Neutrophils (%)	58.4	Unknown	55.6
Lymphocytes (%)	36	Unknown	38.7
CRP (mg/dl)	2.5	Unknown	2.47
AST (U/L)	96	84	93
ALT (U/L)	88	69	67.8
EBV-VCA-IgM	+	–	–
EBV-VCA-IgG/IgA	–	+	+
EBV-NA-IgG	–	+	+
EBV-EA-IgG/IgA	+	+	+
EBV DNA (× 10^5^/copies)	3.2	3.66	1.05
EKG	Unavailable	Normal	Second-degree atrioventricular block
Echocardiogram (mm)	None		
Aortic sinus inner diameter		33.4	31.6
LCA inner diameter		6.4	Widened
RCA aneurysmal dilation		16.6 × 23.6	Widened
Ascending aorta inner diameter		Normal	21.5
Aortic arch inner diameter		Normal	16.4
Descending aorta inner diameter		Normal	19.9
CTA (mm)	None		
RCS aneurysm		22 × 25	Unavailable
LCA inner diameter		Widened	7.9
RCA inner diameter		Widened	3.5
Ascending aorta inner diameter		Mild widened	27.9
Aortic arch inner diameter		Mild widened	21.1
Descending aorta inner diameter		Mild widened	27.7
**Treatments (days)**			
Ganciclovir	14	17	NG
Aspirin	NG	17	8
Warfarin	NG	17	8
Dipyridamole	NG	NG	9
Metoprolol	NG	NG	9

*WBC, white blood cell; CRP, C-reactive protein; AST, aspartate transaminase; ALT, alanine transaminase; VCA, viral capsid antigen; NA, nuclear antigen; EA, early antigen; NG, no given; CTA, computed tomography angiography; LCA, left coronary artery; RCA, right coronary artery; RCS, right coronary sinus*.

## Discussion

CAEBV infection is a rare but fatal disorder resulting from a persistent EBV infection state and usually affects children and adolescents. Regions with high incidence are mostly East Asia countries (9, 10). The clinical features are highly diverse and insidious, with potential involvement of different organs, and its severity varies clinically. Common presentations include intermittent fever, generalized lymphadenopathy, hepatosplenomegaly, liver dysfunction, hypersensitivity to mosquito bites, and rash. Hematologic tests often show anemia, thrombocytopenia, and positive EBV serologic tests. EBV genome can be detected if a biopsy is warranted (2, 11).

Many major complications have been reported. Cardiovascular commodities can occur as an initial manifestation. Among those cardiovascular complications, coronary artery lesions (ectasia or aneurysm) are more common. Other manifestations are cardiac enlargement, valvular regurgitation, pericardial effusion, pulmonary hypertension, cardiac conduction disorders, decreased left ventricular ejection fraction, and sudden cardiac death (3–5, 12, 13). Cardiovascular complication is an important risk factor for mortality in pediatric patients with CAEBV infection (4). As reported by a nationwide survey in Japan, coronary artery aneurysms developed in 9% and myocarditis in 6% of CAEBV patients, and both were associated with death (11). In 1988, Kobayashi et al. first reported one child with CAEBV accompanied by dilation of left and right coronary arteries (14). Up to now, all cases of patients with CAEBV associated with CAL are reported from Japan (4, 6–8, 15) and China (12, 16–18). Our patient in this case had multiple severe cardiovascular complications, her ECG showed second degree of AV block, and the echocardiogram revealed moderate mitral valve and tricuspid regurgitation, mild aortic regurgitation, bilateral SoV aneurysms, bilateral coronary artery lesions, aorta, and its branch dilations. The cause of death 15 months after discharge was presumed to be acute cardiac tamponade due to rupture of the right SoV aneurysm.

CAEBV infection complicated by CAL should be differentiated from Kawasaki disease (KD). Based on the diagnostic criteria published by Kawasaki in 1967, it might not be difficult to differentiate with typical KD (19). However, differential diagnosis of CAEBV infection with incomplete KD might be challenging given the similar presentations. Incomplete KD should be suspected in patients <6 months of age with unexplained fever ≥7 days, even if they have no clinical findings of KD, and in patients of any age with unexplained fever ≥5 days and only two or three clinical criteria of KD (20). Kikuta et al. found a high positive rate of EBV-DNA PCR test of KD patients' peripheral blood samples, which suggests that EBV infection could be one of the etiologies of KD (21). This finding makes the differential even more challenging. In order to identify the reasons for arterial damages, coronary artery tissue should be obtained and sent for EBV-DNA detection. Since it might not be realistic to conduct this biopsy procedure on living patients, we are unable to confirm whether the lesions are caused by EBV infection or KD at this moment (22). Clinical symptoms and follow-up can aid in the diagnostic process. Generally, coronary manifestations of KD usually arise 7–10 days after the initial presentation. However, in CAEBV infection, this can take several years to develop. Of note, many patients lack typical symptoms of KD but present with coronary aneurysms. Given high concerns for rupture, we may treat the patient with intravenous immunoglobulin (IVIG) if incomplete KD is suspected, and the patient's clinical responses and disease progression can help with differentiation. Besides, the ongoing coronavirus disease 2019 (COVID-19) pandemic has aroused global health concerns. Patients with CAEBV infection associated with CAL also should be differentiated from the multisystem inflammatory syndrome in children (MIS-C). Riphagan et al. first reported MIS-C in April 2020 that children infected with SARS-CoV-2 or in close contact with COVID-19 patients presented with persistent high fever and multisystem injuries included coronary artery lesions. However, the incidence is higher in Western countries and rare in Asia (23, 24).

The pathogenesis of cardiovascular lesions in CAEBV infection remains unclear. A possible mechanism is that through infection of T cells, Nature Killer (NK) cells, or B cells, EBV can induce local inflammatory infiltration, cytotoxic injury, and release of cytokines (25). Those processes that produce cytokines such as tumor necrosis factor (TNF)-α will increase the expression of intercellular adhesion molecules on both the EBV-infected leukocytes and the vascular endothelial cells. Compared with EBV-negative cells, EBV-positive cells are more likely to adhere to vascular endothelium and infiltrate into different layers of endocardium, myocardium, and vascular wall. Besides, those EBV-positive leukocytes often exhibit a more potent angiodestructive characteristic and will initiate local vascular lesions, leading to myocarditis or vasculitis, which can further cause arterial dilations and aneurysms (26). Further, some CAEBV patients may initially present with skin ulcers suggesting cutaneous vasculitis, and histopathological examination of a skin biopsy sample from the lesion reveals perivascular lymphocytic infiltration and fibrinoid necrosis of the small vascular wall (5).

Ethnicity is also significantly associated with the prognosis. Compared with patients with CAEBV infection in Asia, patients from Western countries have a generally better prognosis with lower morbidity and mortality. The difference may be related to the type of cells affected (25). As discussed above, T cells and NK cells play a central role in the pathogenesis of CAEBV infection, and involvement of those cells is more common in Asian patients. Cases reported from Western countries are usually associated with proliferation of EBV-infected B cells, with cases of T- and NK cell disease less common. This distribution is analogous to that of extranodal NK/T cell lymphoma, also referred to as nasal NK/T-cell lymphoma, which is also thought to be precipitated by EBV infection (9, 27). More geographical studies are required for this racial distribution.

Therapeutic options for CAEBV infection are limited. Okamura et al. first reported the successful treatment with hematopoietic stem cell transplantation (HSCT) in 2000. Other traditional treatments, including chemotherapy, antiviral, and immunomodulatory drugs, all have limited success (28). In 2011, Kawa et al. performed allo-HSCT for 29 patients with CAEBV, using either myeloablative conditioning (MAC) allo-HSCT (MAST) or reduced-intensity conditioning (RIC) allo-HSCT (RIST). They concluded that the 3-year overall survival rate was 54.5 ± 15.0% for MAST group and 95.0 ± 4.9% for RIST group, indicating that allo-HSCT after RIC seems to be a promising approach for the treatment of CAEBV (29). Recently, Yonese et al. performed a nationwide survey in Japan of pediatric and adult patients with systemic CAEBV. They concluded that the 3-year overall survival rates in patients treated with chemotherapy only, chemotherapy followed by allogeneic hematopoietic stem cell transplantation, and allo-HSCT only were 0, 65, and 82%, respectively (30).

In summary, the prognosis of pediatric patients with CAEBV associated with cardiovascular complications is poor. In the absence of effective therapy, most patients will die of aneurysm rupture or acute respiratory failure in a year. Thus, in pediatric patients with CAEBV infection accompanied by cardiovascular complications, it is crucial to take a thorough history and perform a comprehensive diagnostic workup with the proper EBV serological and genome tests, followed by imaging examinations (echocardiogram or whole-body CT angiogram). An early diagnosis would allow earlier treatment and improve the overall prognosis.

There are limitations to this case report. Due to lack of available clinical information at local hospitals, a *Z*-score was not obtained, increasing the challenges of determining antiplatelet/anticoagulation usage. Evaluation of EBV-infected T or NK cells through lymph node biopsy was not performed due to parents' decline, which makes the diagnosis of CAEBV a strong suspicion based on clinical findings and serologic evidence, but lacking histopathological proof.

## Data Availability Statement

The original contributions presented in the study are included in the article/supplementary material, further inquiries can be directed to the corresponding author/s.

## Ethics Statement

Ethical review and approval was not required for the study on human participants in accordance with the local legislation and institutional requirements. Written informed consent from the participants' legal guardian/next of kin was not required to participate in this study in accordance with the national legislation and the institutional requirements.

## Author Contributions

QL, GL, and DS participated in collecting data and drafted the manuscript. QL provided the case and imaging data. TY and YY gave suggestions for manuscript revisions and participated in its design. All authors contributed to the article and approved the submitted version.

## Conflict of Interest

The authors declare that the research was conducted in the absence of any commercial or financial relationships that could be construed as a potential conflict of interest.

## Publisher's Note

All claims expressed in this article are solely those of the authors and do not necessarily represent those of their affiliated organizations, or those of the publisher, the editors and the reviewers. Any product that may be evaluated in this article, or claim that may be made by its manufacturer, is not guaranteed or endorsed by the publisher.
